# Assessing the effect of high-repetitive single limb exercises (HRSLE) on exercise capacity and quality of life in patients with chronic obstructive pulmonary disease (COPD): study protocol for randomized controlled trial

**DOI:** 10.1186/1745-6215-13-114

**Published:** 2012-07-23

**Authors:** Andre Nyberg, Britta Lindström, Karin Wadell

**Affiliations:** 1 Department of Community Medicine and Rehabilitation, Physiotherapy, Umeå University, Umeå, 90187, Sweden; 2 Department of Public Health and Clinical Medicine Division of Medicine, Umeå University, Umeå, 90187, Sweden

**Keywords:** COPD, Dyspnea, Elastic resistance, Exercise capacity, Multicenter, Quality of life, Randomized controlled trial, Single limb training

## Abstract

**Background:**

Single-limb knee extension exercises have been found to be effective at improving lower extremity exercise capacity in patients with chronic obstructive pulmonary disease (COPD). Since the positive local physiological effects of exercise training only occur in the engaged muscle(s), should upper extremity muscles also be included to determine the effect of single limb exercises in COPD patients.

**Methods/design:**

Trial design: a prospective, assessor-blind, block randomized controlled, parallel-group multicenter trial. Participants: stage II-IV COPD patients, > 40 years of age, ex-smokers, with stable medical treatment will be included starting May 2011. Recruitment at three locations in Sweden. Interventions: 1) high-repetitive single limb exercise (HRSLE) training with elastic bands, 60 minutes, three times/week for 8 weeks combined with four sessions of 60 minutes patient education, or 2) the same patient education alone. Outcomes: *Primary*: determine the effects of HRSLE on local muscle endurance capacity (measured as meters walked during 6-minute walk test and rings moved on 6-minute ring and pegboard test) and quality of life (measured as change on the Swedish version of the Chronic Respiratory Disease Questionnaire). *Secondary:* effects on maximal strength, muscular endurance, dyspnea, self-efficacy, anxiety and depression. The relationship between changes in health-related variables and changes in exercise capacity, sex-related differences in training effects, feasibility of the program, strategies to determine adequate starting resistance and provide accurate resistance for each involved movement and the relationship between muscle fatigue and dyspnea in the different exercise tests will also be analyzed. Randomization: performed by a person independent of the recruitment process and using a computer random number generator. Stratification by center and gender with a 1:1 allocation to the intervention or control using random block sizes. Blinding: all outcome assessors will be blinded to group assignment.

**Discussion:**

The results of this project will contribute to increase the body of knowledge regarding COPD and HRSLE.

**Trial registration:**

ClinicalTrials.gov Identifier: NCT01354067. Registration date: 2011-05-11. First participant randomized: 2011-09-02

## Background

Chronic obstructive pulmonary disease (COPD) is a major cause of chronic morbidity and mortality in the world [1]. COPD was originally a disease more commonly seen in men, but now the disease affects men and women almost equally, as observed in international data [2]. Furthermore, women seem to be more sensitive to the negative effects of tobacco smoke, and develop very severe COPD to a larger extent compared to men [3]. Female COPD patients also demonstrate lower quality of life (QoL), more severe dyspnea and more sensitive airways than men with the same degree of airway obstruction [4]. Exercise intolerance is the key disabling factor in COPD, with decreased exercise capacity, increased leg fatigue and dyspnea among the most frequently reported symptoms [5]. Peripheral muscle weakness has been shown to contribute to exercise intolerance in COPD patients [6]. Nevertheless, Serres and colleagues [7] argued that muscle weakness alone does not explain the decreased peripheral muscle performance seen in patients with COPD. Decreased capacity of local muscle endurance in both the upper and the lower limbs has been shown in COPD patients compared to healthy controls [8]. Different training modalities have been evaluated to uncover the most effective way of training patients with COPD. The primary methods of exercise training within pulmonary rehabilitation have traditionally been different types of exercises incorporating a large amount of muscle mass. However, increased dyspnea during these whole body/large muscle mass exercises cause many COPD patients to stop exercising before their cardiovascular system or skeletal muscles are maximally stressed [9]. Training using a reduced muscle mass is a way of dealing with this issue. This has been found to achieve a higher metabolic rate due to less stress being placed on the respiratory system as ventilation is decreased compared to whole body exercises [10]. For example, three recent studies have shown positive effects of single limb training (SLT) (that is, one-legged cycling/knee extensor training) in patients with COPD [11-13]. Two of these studies [11,12] focusing on one-legged cycling concluded that it was superior to two-legged cycling regarding aerobic capacity for COPD patients. Bjørgen and colleagues [11] also demonstrated that the total amount of work was larger in the group working with one leg at a time compared to the group working with both legs simultaneously. This could be somewhat explained by an increased capacity of local muscle endurance due to increased oxygen uptake and increased maximal mitochondrial respiration in working muscles, as demonstrated in the one-legged knee extensor training study by Brønstad and colleagues [13].

High-repetitive SLT (that is, exercising only one arm or one leg at a time) using elastic resistance bands on the upper and lower extremity muscles within an exercise regimen have previously been used with success in patients with chronic heart failure [14]. Elastic bands as resistance have also been used in patients with COPD [15], however the concept of incorporating upper as well as lower extremity muscles within an SLT regimen has not been tested in patients with COPD. A goal-based multiple repetitions maximum (RM) test to obtain the accurate load using elastic bands has previously been used, though this is time consuming and could be anticipated to take between three and six attempts [16,17] for each separate exercise. As far as we know, there are no published studies investigating the effect of local high-repetitive single limb exercises in patients with COPD. In addition, because of the enhanced number of women affected by COPD [2], a better understanding of the effects of training in women is of importance. Previous studies on SLT have only incorporated the leg muscles (quadriceps in particular) [11-13]. To be able to optimize training for all COPD patients while focusing on local muscle endurance, a clinical trial incorporating upper as well as lower extremity muscles is essential. In addition, if the starting resistance is accurate for each targeted muscle the amount of time exercising with adequate resistance will increase.

## Methods/design

**Figure 1:**
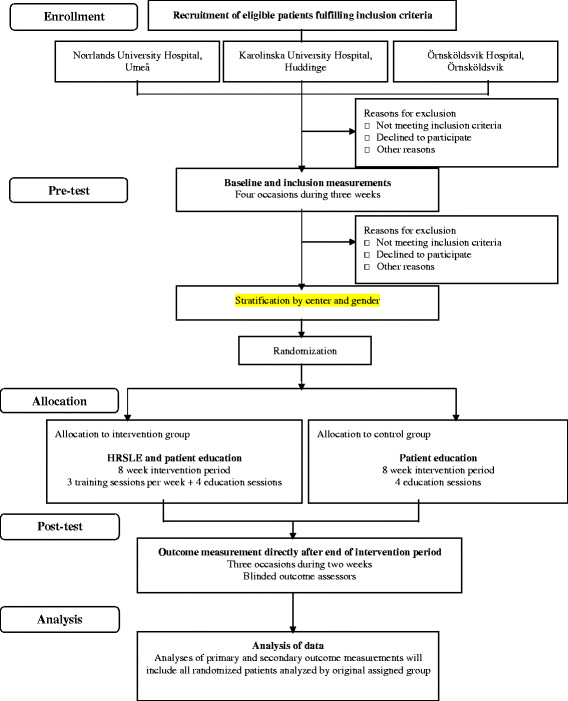
**Consolidate standards of reporting trials** (**CONSORT) flow diagram of trial design.**

This is a prospective, assessor-blind, block randomized controlled, parallel-group multicenter trial constructed in accordance with consolidate standards of reporting trials (CONSORT) [18,19] guidelines (Figure 1).

### Objectives

The aim of the current randomized controlled multicenter trial (RCT) is to determine the effects of high-repetitive single limb exercises (HRSLE) in combination with COPD-specific patient education, compared to the COPD-specific patient education alone in patients with moderate to very severe (stage II-IV) COPD.

#### Primary outcomes

1. Lower extremity: determine the effects of HRSLE on local muscle endurance capacity measured as meters walked during 6-minute walk test (6 MWT).

2. Upper extremity: determine the effects of HRSLE on local muscle endurance capacity measured as rings moved on 6-minute ring and pegboard test.

3. Determine the effects of HRSLE on QoL measured as the change in score on the Swedish version of the chronic respiratory disease questionnaire (CRQ-SAS).

#### Secondary outcomes

1. Determine the effect of HRSLE on: 1) endurance time, ventilatory response, perceived dyspnea and leg fatigue during submaximal cycle test; 2) maximal strength and muscular endurance during isokinetic tests; and 3) self reported dyspnea, self-efficacy, anxiety and depression.

2. Determine the relationship between: 1) changes in self reported dyspnea, self-efficacy, anxiety and depression; and 2) changes in exercise capacity

3. Identify if there are any sex-related differences in exercise training effects.

4. Determine if this exercise regimen is feasible and safe in stage II-IV COPD patients.

5. Develop a strategy to minimize attempts needed to determine a multiple repetition resistance using elastic bands.

6. Develop a strategy to optimize resistance for each involved movement within an exercise regimen.

7. Investigate the relationship between muscle fatigue and dyspnea in the different exercise tests.

### Settings

The trial is conducted at three hospitals in Sweden: one in the county of Västerbotten (Norrlands University Hospital, Umeå), one in the region of Stockholm (Karolinska University Hospital, Huddinge) and one in the region of Västernorrland (Örnsköldsvik Hospital, Örnsköldsvik). Recruitment of participants and collection of data started May 2011.

### Eligibility criteria

Potentially eligible participants are being identified through screening of patient medical records at participating hospitals as well as medical records from primary care centers in the vicinity of each hospital. Inclusion criteria are verified through information obtained from patient medical records in three steps.

(1) Screening is performed by one of the authors (AN) who divides screened medical records into three groups: (a) eligible, (b) potentially eligible, and (c) excluded.

(2) Those found eligible or potentially eligible are controlled and verified by a second author (KW) or by pulmonary medical doctors at respective hospital.

(3) If missing or unclear data is present in patient medical records, information regarding inclusion criteria is obtained from those found eligible through standardized questions during phone contact at the recruitment process.

### Inclusion criteria

1. Adults 40 years and above.

2. Stable (no exacerbations within 4 weeks before start of baseline testing) moderate to very severe COPD, stage II-IV, according to GOLD criteria [1] (that is, forced expiratory volume in one second/forced vital capacity < 0.70, forced expiratory volume in one second < 80% predicted).

3. Ex-smoker.

4. Stable medical treatment (no changes < 4 weeks before start of baseline testing).

5. Living less than 60 km from training facility.

### Exclusion criteria

1. Musculoskeletal, rheumatic, cardiac or neurological disorders that might affect the exercise performance in training and tests.

2. Previous lung surgery.

3. Acute exacerbations of COPD that require a change in pharmacological management within 4 weeks preceding the start of the intervention.

4. Long-term oxygen treatment.

5. Participated in organized exercise training, > 2 times a week, within 6 months before start of intervention.

6. Body mass index < 18 kg/m^2^.

The following eligibility criteria will be used for the local physiotherapists leading the exercise intervention and giving the COPD-specific patient education.

### Physiotherapist inclusion criteria

Professional qualities for those leading exercise intervention

(1) At least 2 years experience of working as a physiotherapist.

(2) At least 1 year experience of working with COPD patients or 1 year with heart failure patients.

(3) At least 1 year experience of leading training groups.

Professional qualities for those leading patient education

(1) At least 2 years experience of working as a physiotherapist.

(2) At least 1 year experience of working with COPD patients.

(3) Previous experience of leading patient education or pulmonary rehabilitation for COPD patients.

Physiotherapists not fulfilling the above criteria will be excluded.

#### Physiotherapist education

All physiotherapists leading the HRSLE will receive individual standardized education before the start of intervention. The education lasts for 60 minutes and consists of written, oral and visual instructions on how to perform and instruct each exercise within the HRSLE. It also includes instructions on how to progress exercise with the use of Borg CR10 [20], and how to standardize exercises and positions during warm-up, training and cool-down within the HRSLE regimen.

The written information includes:

(1) A check-list for all sessions on what to do, before, during and after each session (including placement of equipment, exercise order, when to rate muscular fatigue and dyspnea with Borg CR10, and use of pulsoximetry).

(2) A comprehensive exercise protocol (see Additional file 1).

(3) Rating sheets for muscular fatigue, dyspnea and saturation.

(4) The written information given to participants in:

a. The intervention group

b. The control group

### Description of intervention and comparator

#### Intervention treatment

The HRSLE regimen consists of three sessions per week (Monday, Wednesday and Friday at approximately noon) during 8 weeks of exercise training, giving a total number of 24 sessions. The HRSLE regimen is supervised and conducted by local physiotherapists using a group format, with four to eight participants in each group. Each session will span approximately 60 minutes duration for the major components: 10 minutes warm-up followed by 40 minutes of HRSLE, and then 10 minutes cool-down.

#### Warm-up

The warm-up focuses on low intensity dynamic flexibility exercises of involved muscle groups to ensure adequate range of motion, familiarization of exercises movements and execution of motions at optimal velocity to rehearse desired motion patterns as recommended by the American College of Sports Medicine (ACSM) [21]. The following warm-up exercises will be performed alternately left to right arm/leg.

Seated: heel raises, knee extension with heel touching floor, hip flexion, rear deltoid row, chest press, knee extension with dorsal flexion of ankle, and biceps flexion with vertical shoulder press.

Standing: walking, two-leg heel raises, hip flexion, single leg anterior/posterior swing, knee extension with heel touching floor, and leg curl.

#### High-repetitive single limb exercises using elastic bands

##### Exercise selection and equipment

Important upper and lower extremity muscle groups for COPD patients have been identified through a literature search focusing on muscles with decreased strength/endurance compared to healthy individuals, upper extremity activities important for daily living, and those important for walking [22-25]. Exercises have been identified for each of the relevant muscle groups. Execution of each exercise is standardized to optimize activation of targeted muscles (for specific details see Additional file 1[6,26,27]). The exercise regimen will consist of eight exercises, of which six single arm/leg exercises (primary muscle focus) [22-25] - latissimus row (m. latissimus dorsi), chest press (m. pectoralis major, m. deltiod anterior), leg extension (m. quadriceps), straight arm shoulder flex (m. deltoid anterior), leg curl (m. hamstrings) and elbow flexion (m. biceps brachii) - are performed with elastic resistance bands, so-called Thera-Bands (The Hygenic Corporation, Akron, OH, USA). The elastic bands are fastened at the lowest and highest levels of wall bars present in training facilities. Eight different Thera-Bands with increasing resistance will be used in this trial: tan (extra thin) < yellow (thin) < red (medium) < green (heavy) < blue (extra heavy) < black (special heavy) < silver (super heavy) < gold (maximum resistance). This allows suitable resistance for each individual and a graded increase in work load during exercises [14]. A change from one color to another results in an increased resistance of 20 to 30% [28]. The two other exercises, single leg heel raise (m. triceps surae) and single leg step-up (m. quadriceps, m. iliopsoas), are performed with body weight as the primary resistance. Elastic resistance is incorporated when progression occurs.

##### Volume and load

Unilateral exercises with high repetitions are selected for this trial in line with recommendations of the ACSM [29]. Unilateral exercises are chosen to minimize the involved muscle mass to customize the intervention for the patients with COPD. High-repetition exercises are also recommended both by Ebben and colleagues [30], for untrained individuals to improve endurance, and by Campos and colleagues [31], who found significant increases in aerobic power and time to exhaustion after high-repetition exercises (20 to 28 repetitions, 1 minute rest).

##### Rest periods

The rest period chosen in this trial is 1 minute, as previously used [31] and recommended when the number of repetitions exceeds 15 for local muscle endurance [29].

##### Frequency

The exercise regimen will be performed 3 days per week [29].

##### Repetition velocity

Moderate to fast velocities are recommended when performing a larger number of repetitions (that is, at least 15 repetitions). In this trial, we will execute 25 repetitions with 1 second in the concentric contraction phase and 1 second in the eccentric contraction phase. This means that we can keep the rest intervals within 1 minute as recommended [29].

##### Progression

The resistance for each exercise will be individually determined and progressed to optimize individual tailoring of the exercise regimen. Progression will be done separately for the six exercises using elastic resistance, the single leg-heel raise and the single leg step-up. Progression will be executed with the use of individual ratings of muscle fatigue and dyspnea according to the Borg CR10 scale [20] following a specified protocol: if a patient rates < 4 on muscle fatigue [32] and performs ≥ 20 repetitions [29] in at least three of six elastic resistance exercises it will result in an increase in resistance. However, irrespective of muscular fatigue, a rating of at least three out of six exercises > 5 on dyspnea [33] will result in a decrease in resistance. The same principle will be used on the single leg step-up and single leg heel raise individually. The Borg scale has previously been used on COPD patients after single leg exercises to measure dyspnea and muscular fatigue [34] and when rating exercise intensity using elastic resistance tubing [35]. To progress, the criteria for rating should be fulfilled in two subsequent sessions or two out of three following sessions. Progression in the six exercises with elastic resistance band is executed by increasing the tension of the elastic band (for example, tan < yellow < red < green < blue < black < silver < gold). Progression of single leg heel raise is as follows: (1) heel raises on flat surface, (2) heel raises on step-up board (allowing larger range of motion), (3) heel raises on step-up board with tan elastic resistance (fastening resistance band under step-up board stretched to the lowest position on the side of the exercising leg), and (4) heel raises on step-up board with increased elastic resistance as described above. Progression of step-up is as follows: (1) 15 cm step-up board, (2) 20 cm step-up board, (3), 25 cm step-up board, (4) 25 cm step-up board with tan elastic resistance (fastening resistance band under step-up board stretched to the lowest position on the side of the exercising leg), and then (5) heel raises on step-up board with increased resistance as described above.

##### Initial load

To be able to enhance the accuracy of each individual patient receiving adequate initial resistance for each separate exercise we aim to use a three-step approach:

1 A pilot study was performed on 30 healthy individuals, in a similar age group as the patients within this study. The results of the pilot study showed a strong relationship between elastic resistance maximal voluntary contraction (MVC) and isokinetic peak value during shoulder flexion with no significant difference in weight (kg). Based on this pilot study, the peak value obtained from the isokinetic tests at baseline within the present study will be used to determine MVC (that is, 1 RM) using elastic resistance [36]. Fifty-five per cent of the isokinetic peak value will be used as 25 RM as recommended by Page and Ellenbecker [36]. However, the choice of standardizing the training load from a MVC using elastic resistance has been questioned. Therefore, a goal-based 25 RM test will also be performed [37]. The number of attempts needed to reach 25 RM will be recorded.

2 Based on a literature search the following percentage strength relationships between involved muscles for COPD patients during the movements that are involved in this HRSLE were identified (described as weight at end position if knee extension is seen as maximal): knee extension (100%), chest press (85%), leg curl (65%), elbow flexion (50%), latissimus row (35%), shoulder flexion (30%). For example, if 10 kg is considered 55% of 1 RM for knee extension, 3 kg would be considered 55% of 1 RM for shoulder flexion.

3 According to the strength relationship between involved muscles and information obtained regarding the weight properties of elastic resistance at a given length (Thera-Band values provided by the manufacturer; The Hygenic Corporation), the distance from insertion for each exercise using elastic resistance is determined (see Additional file 2).

By using different starting positions for each exercise (that is, the elastic tubing is stretched to different lengths resulting in different resistances at every position) we hope to be able to optimize the resistance for the involved muscle during each separate exercise. Two elastic resistance bands (the same color) will be needed (one fastened at the lowest level of the wall bar and the other fastened at the highest level) for all elastic exercises within the HRSLE.

#### Cool-down

Cool-down will consist of five stretching exercises for involved muscle groups. Holding each stretch for 30 seconds to mild discomfort, with two repetitions for each limb (that is, 2 minutes active stretching for each muscle), targeting the pectoralis major, biceps brachii, hamstrings, quadriceps and triceps surae, is in accordance with recommendations of the ACSM [38]. Selection of muscles is based on involvement in the HRSLE regimen. In addition, the intervention group receives standardized patient education on four occasions during an 8-week period. Each session lasts 60 minutes, and consists of information regarding anatomy, physiology, COPD (causes and mechanisms), nutrition, aids/tools and energy saving procedures.

##### Standardization

To enhance repeatability of this HRSLE regimen, the following key elements are being standardized:

The length of each individual elastic band is between 3 and 3.5 m (depending on color). The different colors result in different lengths needed for handles and insertion - for example, 1 to 1.5 m is used for insertion and handles and the 2 m remaining results in 1 m to the left and right limb respectively. The relaxed length of the elastic resistance bands from insertion are, as a consequence, standardized to 1 m for each side, pre-stretched 20 times to stabilize the material [39].

Individual starting resistance (color of elastic resistance band) is standardized from isokinetic baseline isokinetic peak value in shoulder flexion and then altered for other exercises according to the strength relationship between the involved muscles for each respective participant.

Distances from elastic resistance insertion for each exercise are standardized, with different colored cones, to optimize adequate resistance for targeted muscle groups. The distance away from insertion for each exercise is based on a literature search of strength relationships between involved muscles for COPD patients and information obtained from the manufacturer regarding the properties of elastic resistance (Additional file 2).

The elastic resistance has been tested for 5,040 cycles with less than 1.1 Newton change in force-generating potential [39]. To obtain the same force-generating potential of the elastic resistance, the bands are changed every eight sessions using a consistent color used based on the following calculations: each exercise is 25 repetitions twice for each extremity = 100 repetitions per exercise, with elastic resistance for six exercises = 600 repetitions per session; 600 times 8 sessions = 4,800 repetitions.

Start and stop positions are standardized for each exercise and executed to optimize activation of targeted muscles for each exercise separately.

The most clinically effective degree of elastic resistance is 25% to 250% elongation; all exercise start and stop positions are within this interval [36].

Individual progression is standardized using Borg CR10 according to a specified protocol (see Progression section above for more detail). For specific details of progression for each exercise see the HRSLE section above.

The targeted velocity - that is, 1 s concentric, 1 s eccentric (60 beats/minute) - for seven out of eight exercises is standardized by counting by the physiotherapist leading the training sessions. In addition, music at twice the pace (around 120 beats/minute) will be used to assist the physiotherapists and patients.

The standardized exercise protocol (Additional file 1) will be given to both participants and physiotherapists to enhance adherence and familiarization.

Patient education material is standardized and the same for all locations.

Physiotherapists are given standardized education to uniformly deliver the intervention.

In addition to the exercise protocol, standardized instructions on exercise progression and distance from elastic resistance insertion for each exercise (as described above) will be given to the physiotherapists leading the training to enhance adherence. Furthermore, written commitments to follow the assigned protocol will be signed by all physiotherapists and adherence to trial protocol will be evaluated by a questionnaire after the end of intervention for both participants and physiotherapists.

#### Comparator

The control group receives standardized patient education four times during the 8-week intervention period. Each session lasts 60 minutes, and consists of information regarding: anatomy, physiology, COPD (causes and mechanisms), nutrition, aids/tools and energy saving procedures. The information is the same as for the intervention group, but given on separate occasions. After post-tests, the participants in the control group will be offered participation in a training intervention.

##### Standardization

To enhance repeatability of patient education, the following key elements are being standardized:

Patient education material is standardized and the same for all locations.

Physiotherapists give informed consent to follow the standardized instructions regarding the patient education materials.

### Screening tests and outcome assessment

Different tests will be performed before the start of the study and directly after the 8-week intervention period at Norrlands University Hospital, Umeå, and Karolinska University Hospital, Huddinge. Participants at Örnsköldsvik Hospital, Örnsköldsvik, will perform tests at Norrlands University hospital, Umeå, due to lack of specific test equipment. The primary outcome measures will be assessed by the same researcher (AN) for all participants at each location. Pre-tests will be performed on four occasions, within 3 weeks, before the start of intervention, and post-tests will be performed on three occasions within 2 weeks. Each test occasion will last from 1 to 2 h. Pre- and post-tests will be performed at the same time of day, with the same rest between test occasions and during tests. Since a subject's motivation may determine attainment of maximal effort [7], both pre- and post-tests are performed using standardized information and encouragement [32]. No additional follow-up apart from directly after the end of the intervention period is used within this trial. However, previous studies show that the benefits of exercise training on exercise capacity and QoL in patients with COPD appear to decline by 12 to 24 months despite different exercise strategies [40,41]. There is an urgent need to find effective strategies on how to maintain the positive effects for a longer period. Therefore, in connection with the study described in this protocol, a randomized controlled study is planned to evaluate the effect of motivational interviewing as a strategy to maintain/increase the level of physical activity after a training program (and by that also the benefits achieved from the training program). Participants randomized to the intervention group will be asked to participate, and follow-up tests will be performed at 6, 12 and 24 months. A trial protocol will be published with further description.

### Screening tests

#### Pulmonary function testing

Spirometry, body plethysmography, and single-breath diffusing capacity maneuver will be performed using the Jaeger Masterscreen Body (CareFusion, GmbH, Hoechberg, Germany) at Umeå University Hospital or at Huddinge University Hospital in accordance with recommended techniques [42,43].

#### Cardiopulmonary exercise testing - incremental

Symptom-limited exercise tests will be conducted on an electronically-braked cycle (Rodby RE 990 1063, Hagby, Sweden) at the Department of Clinical Physiology, Umeå University, and at Karolinska University Hospital, Huddinge, in accordance with clinical exercise testing guidelines [44]. An incremental exercise test with work rate increments of 10 Watts per minute until symptom limitation will be performed. Patients will rate their dyspnea and leg fatigue according to the Borg CR-10 scale [20] every minute during the test. Watts maximum (W_max_) is defined as the greatest work rate that the subject is able to maintain for at least 30 s.

### Outcome measures

#### Cardiopulmonary exercise testing – constant work rate

Constant work rate exercise tests will be performed at 75% W_max_ (obtained from cardiopulmonary exercise testing (CPET) – incremental test). Endurance time will be recorded as the time from the increase in work rate to 75% W_max_ to the point of symptom limitation. During the constant-load exercise test, measurement of ventilation, oxygen uptake and carbon dioxide production will be measured by ergospirometry (VMAX Encore 229, CareFusion, Palm Springs, USA in Huddinge and Jaeger Oxycon, CareFusion, GmbH, Hoechberg, Germany in Umeå). CPET is considered the gold standard for measuring the level of exercise limitations of a patient and is often used in patients with COPD [45].

#### Field test

The following tests will be performed at the department of Community Medicine and Rehabilitation, Physiotherapy, Umeå University, and at the Physiotherapy division in Karolinska University Hospital, Huddinge: dyspnea and muscle fatigue in the legs and arms, depending on the test, will be measured with the Borg CR10 [46], and oxygen saturation and pulse will be measured with pulsoximetry (Nellcore NPB-40, Pleasanton, CA, USA) before and directly after each test. The Borg CR10 scale has previously been used to evaluate dyspnea, breathlessness and leg discomfort in COPD patients [47] and is found to be reliable [48].

##### Upper extremity

*Primary outcome:* To assess functional muscular capacity in the upper extremity, the 6-minute pegboard and ring test will be used [49]. The pegboard and ring test consists of a peg board with the pegs set at the subject’s shoulder height and 10 cm below shoulder height. The pegs will be at shoulder width; three different insertions are available (45, 50 and 55 cm width) to be suitable for all participants. The rings consist of 10 lightweight wooden rings (6 g each) that the subject moves simultaneously from the upper pegs to the lower pegs repeatedly [50]. The subjects are asked to move as many rings as possible in 6 minutes and the final score is the total number of rings moved in the given amount of time. Standardized encouragement will be given to the subjects each minute. All subjects will move 20 rings from the lower to the upper pegs and reverse prior to the test to familiarize themselves with the test procedure and lower the risk for learning effect. The 6 minute pegboard and ring test has previously been used on COPD patients [51] and shows excellent test-retest reliability [49]. To measure unsupported endurance capacity, the Unsupported Upper Limb Exercise test (UULEX) will be used [52]. The patients hold a plastic bar (0.2 kg) at shoulder width. The UULEX consists of eight levels; the patients perform each level for 1 minute, lifting the plastic bar at a cadence of 30 beats per minute, controlled with a metronome, from the hip to the UULEX eight level chart. The tests starts with a 2-minute warm up at the lowest level; the warm up is included in the final time. The first level is adjusted to be at the level of the patient’s knees by altering the position of the UULEX chart on the wall. Once they have reached their maximum height the bar will be replaced with a heavier one (0.2 < 0.5 < 1 < 1.5 < 2 kg). The patients are instructed to perform the test until symptom limitation. The UULEX has previously been used on COPD patients [51].

##### Lower extremity

*Primary outcome:* The 6 MWT will be used to assess lower extremity functional muscular endurance and walking capacity. The 6 MWT is widely used on COPD patients for measurement of endurance walking capacity [53]. In this study we expect to see a difference of 50 m based on a recent published RCT on unsupported training and COPD [54]. The walking course will be 30 m in length and the patients will be instructed in accordance with standardized guidelines [55] to walk as far as possible in 6 minutes. One practice test will be performed to minimize risk of learning effect. The highest value will be chosen as the baseline value.

#### Peripheral muscle strength

Isokinetic muscle strength (maximal and endurance) in the leg (knee extension) and the arm (shoulder flexion) will be evaluated by the use of two interchangeable stationary dynamometers: the Biodex Multi-Joint System 3 (Biodex Corp., Shirley, NY, USA) at the Department of Community Medicine and Rehabilitation, Physiotherapy, Umeå University; and The Biodex Multi-Joint System 4 (Biodex Corp., Shirley) at Karolinska University Hospital, Huddinge. The tests will be performed unilaterally on the self-reported, dominant side. Subjects will be seated in accordance with the manufacturer’s recommendations. The 6 MWT is performed before the isokinetic tests and will be considered as a warm-up. Before both maximal tests the participants will perform five submaximal contractions to familiarize themselves with the equipment and testing procedure, followed by 2 minutes standardized rest before the start of testing [32].

##### Shoulder flexion

Test of shoulder flexion will be performed between 0° and 100°; great care will be taken to align the glenohumeral joint with the movement axis of the Biodex dynamometers (The Biodex Multi-Joint System 3 and 4 (Biodex Corp., Shirley)). The arm will be held with the elbow extended and the forearm semi-pronated [56].

##### Knee extension

The test of knee extension will be performed between 90° flexion to maximal knee extension −5°. Full extension −5° will be used to lower the risk for a passive resistance from the hamstring muscle during knee extension. Great care will be taken to align the lateral femur epicondyle with the movement axis of the Biodex dynamometers (The Biodex Multi-Joint System 3 and 4 (Biodex Corp., Shirley)). Positioning of participants, equipment, and range of motion will be documented to be the same during pre- and post-tests. Two different aspects of isokinetic muscle strength will be measured. Firstly, a maximum contraction test measuring peak torque (Nm) from the highest contraction at an angular velocity of 60°·per s, generated through five maximal contractions, will be performed as previously used and recommended for COPD patients [57-59]. The subjects are told to flex their arm or extend their leg five times in a row with maximal effort during the concentric phase and to rest during the eccentric phase. Secondly, after a recovery period of 5 minutes [32,59], an endurance test consisting of 30 consecutive repetitions [59] at 60°·per s measuring total work (joules) will be performed. The subjects are told to flex their arm or extend their leg 30 times using maximal effort at each repetition during the concentric phase and to rest during the eccentric phase. The angular velocity of 60°·per s is considered ideal for evaluating muscle strength in COPD patients [57] and is found reliable in endurance tests [60]. No visual feedback is given during tests. Standardized encouragement is given.

#### Chronic dyspnea questionnaires

The Medical Research Council (MRC) Dyspnea scale is a five-point scale based on degrees of various physical activities that cause dyspnea [61] and will be used to grade the patients’ inconvenience. The MRC dyspnea scale has been used in numerous clinical trials involving COPD [62], and is shown to be valid in COPD patients [63].

#### Health-related quality of life

##### Primary outcome

The CRQ-SAS version of the CRQ [64] will be used to assess QoL. The CRQ is a widely used disease specific questionnaire to assess symptoms of COPD patients [65]. The Swedish CRQ-SAS consists of 20 health-related questions. The Swedish version of the Clinical COPD questionnaire (CCQ) will also be used to assess disease-specific QoL [66]. The CCQ has been shown to be valid and reliable in Swedish patients with COPD [67] and consists of 10 questions. The Swedish SF-36 will be used for the assessment of QoL. SF-36 is a 36-item questionnaire used to evaluate QoL through eight subscales: physical functioning, role-physical, role-emotional, social functioning, general health, mental health, bodily pain and vitality [68]. The SF-36 has previously been used in the assessment of QoL in COPD patients [69] and is considered the gold standard generic health assessment tool [70], being both valid, reliable and vigorously tested [68].

#### Anxiety, depression and self-efficacy

The Hospital Anxiety and Depression Scale (HADs) will be used for assessment of anxiety and depression. HADs consists of 14 items, producing separate scores for anxiety and depression [71]. The questionnaire is widely used in association with COPD [72]. The Swedish version of the Exercise Self-Efficacy Scale (ESES) [73] will be used to investigate self-efficacy. The ESES consists of 10 questions and the Swedish version is now undergoing validity and reliability tests.

The Self Efficacy for Walking Questionnaire will be used to assess the patients self efficacy in association with physical activity, and the questionnaire has been used in intervention studies on patients with COPD [74].

#### Feasibility

Feasibility will be evaluated through attendance, adherence and development of any side effects or injury during each training session. In addition, compliance to the different parts of the HRSLE will be evaluated with a standardized questionnaire by both study participants and the physiotherapists leading the interventions.

#### Training resistance

To determine starting resistance (color of resistance band) a 25 RM shoulder flexion test will be performed, standing using the dominant arm, during baseline tests. The exercise movement will be instructed orally and visually by the primary investigator (AN). The following familiarization strategy will be used: 25 repetitions will be performed with the non-dominant arm using the lowest level of resistance (tan elastic band) to familiarize the patient with the exercise movement [37]. During familiarization, the patient will receive instructions to perform the exercise accurately - arm kept straight (elbow extended) throughout the whole movement holding the band in full can position with thumb pointing upwards; lifting the arm upwards/forward until the elbow passes the patient’s chin returning to the start position in a controlled manner with the same speed [27]. Length of the elastic band will be 1 m, stretched 25% at the start and proximally 75% at the end position, that is 1.75 m at the end position. During the test the patient will be instructed to perform as many repetitions as possible, and no additional feedback or encouragement will be provided. The 55% of peak value obtained during isokinetic shoulder flexion will be obtained and the elastic resistance band closest to this value at 75% elongation will be used. A change of elastic band results in a weight difference of 20 to 30% [28], and between 20 and 30 repetitions are considered 25 RM. The number of attempts needed to reach 25 RM will be recorded. Five minutes rest between attempts will be provided if necessary. Rating of muscle fatigue and dyspnea after each exercise using BORG CR10 will be recorded for each participant randomized to the intervention group.

### Sample size

A sample size of 17 in each group (intervention, control) will be required to obtain the power to detect a significant difference in lower limb local muscular endurance capacity measured by the 6-MWT [53]. The sample size calculation was made with the following assumptions: a relevant mean difference of 50 meters walked in 6 minutes with a standard deviation (SD) of 52, α = 0.05, β = 0.20 (80% power); and a two-tailed test of significance, based on a study by Costi and colleagues [54] (a RCT published in 2009 on unsupported training in patients with COPD). For QoL, the sample size calculation was made with these assumptions: a relevant mean difference of 1 unit (SD 1.07) on the Swedish version of the CRQ, α = 0.05, β = 0.20 (80% power); and a two-tailed test of significance, data based on CRQ values obtained in the study by Lacasse and colleagues [75] gave 17 patients in each group. Multicenter trials involve a correlation in data - subjects from the same center are likely to be more similar than subjects from other centers. Because such a correlation potentially affects the power of the data within the trial, the sample size needs to be adjusted, often increased, to account for the design of the study - in other words, the design effect [76]. Several considerations have been made to minimize the effect of using different centers: the same inclusion criteria for participants at all locations; specified and similar inclusion criteria for physiotherapists at all locations; standardized training for all physiotherapists to uniformly deliver the intervention; all aspects of the intervention regimen are standardized; a standardized exercise protocol will be given to both physiotherapists leading interventions and participants; physiotherapists give informed consent to follow the standardized instructions regarding the standardized patient education material; and exercise protocol and primary outcome measures are measured by the same (blinded) outcome assessor at all centers during pre- and post-tests. In addition, in an individually randomized trial, stratified for centers, the design effect is estimated to be < 1 which involves a gain in power, allowing a reduction in sample size rather than an increase [76,77]. With regard to the design of the study and the considerations taken to minimize the effect of using different centers, no adjustment of sample size will be made due to design effect. Nevertheless, the sample size is adjusted for potential drop-outs. We expect a drop-out rate of 20% based on a previous systematic review [78]. We aim to recruit five additional patients to each group, resulting in a total number of 44 patients, 22 in each group (17/0.8 = 21.3 ≈ 22) with moderate to very severe COPD. The total study group will consist of an equal number of males and females.

### Randomization and blinding

To prevent knowledge of treatment assignment and to keep the allocation sequence concealed from the participants and the researchers, group allocation will be completed after baseline and the inclusions tests performed by an individual independent of the recruitment process. This person will, after enrollment of participants, be emailed participation details and then perform the randomization using a randomly permuted blocks design with a computer random number generator (http://www.randomization.com). Randomization will be stratified by center and gender with a 1:1 allocation to the intervention or control group using random block sizes. The assignment to groups after randomization will be performed by a third party independent of the recruitment process for allocation and randomization. After randomization the allocation sequence will be kept in an opaque, sealed and stapled envelope, locked away in a safe deposit box at the department of Community Medicine and Rehabilitation, Physiotherapy, Umeå University, Umeå, and will be kept concealed until the end of outcome assessment. The envelope will be made impermeable to intense light by using aluminum foil inside and sealed using tamper-proof labels. Furthermore, the participants will be given repeated instructions not to reveal their group allocation to the outcome assessors during post-tests after the end of the intervention period. In case of a failure in keeping the outcome assessor blinded (that is, a patient reveals his/her group allocation) a second trained outcome assessor will be available to step in and conduct the post-tests.

### Statistics

The analysis of primary and secondary outcome measurements will include all randomized patients. To be able to use all patient data, the mixed models approach will be adopted with individuals at level one and center at level two. The analysis will demonstrate any improvements at follow-up, comparing the intervention and control group. In linear mixed models the variable group is considered main, controlling for baseline value, center and gender. To judge the quality of the model we will analyze the residuals. A difference at the *P* < 0.05 level will be considered significant in all outcome measures and analyses. For each outcome the results will be reported as a summary of the outcome in each group presented as mean and SD, together with effect size. For treatment effect, 95% confidence intervals (CI) and *P* values will be presented. For each group, information about the number of participants included in each separate analysis and whether theses analyses were by original assigned groups will be given. Correlation between changes in physical variables and changes in health variables will be measured by Pearson correlation. A pre-specified subgroup analysis of participants randomized to the intervention group will be performed to investigate differences in BORG CR10 rating between elastic resistance exercises to evaluate the strength relationship between involved muscles/movements. Our research group has previously found relatively lower thigh muscle strength in female patients with COPD compared to male patients with COPD and healthy controls [32]. In addition, Skumlien and colleagues [79] found gender differences regarding effects following a pulmonary rehabilitation program and further research regarding the potential differences in training effects between female and male patients with COPD is required. Therefore, we aim to compare primary and secondary outcome measures for males versus females in pre-specified subgroup analyses to gather information for future studies. Intention-to-treat analysis will be used for all outcome measurements and involve all patients who are randomly assigned. Mixed models will be used to handle missing outcome data. In addition, within the main analysis, an on-treatment analysis will be executed. On-treatment is defined as at least 80% compliance - in other words, at least 20 training sessions or three education sessions depending on group allocation. For data management and statistical analysis the IBM Statistical Package for Social Sciences (SPSS) version 20.0 will be used.

### Ethical approval and informed consent

The study has been approved by the regional ethical board, Umeå, Sweden. Dnr: 2010-344-31 M. All participants will receive brief and comprehensible oral and written information, in accordance with the Helsinki declaration [80]. Informed, written consent is obtained from all participants by one of the researchers (AN) before baseline tests.

## Discussion

The HRSLE trial is, to our knowledge, the first trial investigating the effect of high-repetitive single limb exercises in moderate to severe COPD patients. This is a well designed study in strict accordance with CONSORT guidelines, which will give us the opportunity to determine the effects of the HRSLE regimen used within this trial. A problem in many previous exercise studies is the lack of standardization which makes reproducibility difficult. The comprehensive standardization in this trial facilitates the reproducibility of this trial. There is evidence that a higher metabolic rate is achieved by training using a reduced simultaneous muscle mass in the form of single limb training (that is, training using one leg/arm at a time) in COPD patients. This is due to less stress being placed on the respiratory system as ventilation is reduced compared to whole body exercises [10,81]. Previous studies indicate that single limb exercise regimens seem more effective regarding total work conducted during exercise compared to using a larger amount of muscle mass [11,12]. This project will give important information about a single limb exercise regimen that gives patients the opportunity to incorporate both upper as well as lower extremity muscles within the same exercise regimen maintaining a low simultaneous impact on the ventilatory system. To find adjunctive exercise strategies is of great importance to optimize the treatment of the growing population of COPD patients. The results of this project will most likely influence how we treat COPD patients in the future. The number of females with COPD is increasing but most of the previously conducted exercise trials involve a majority of men. It is very important to investigate if the treatment effects differ between men and women to be able to optimize the treatment for all patients suffering from this troublesome disease. This RCT will contribute to the existing knowledge regarding potential sex-related differences in physiological response to exercise training. Adequate resistance for each separately involved muscle within an exercise regimen is of importance to optimize the training effects. If we could minimize the attempts needed to reach adequate resistance and provide accurate resistance for each involved muscle the effects of the exercise training could increase.

### Trial status

The trial is currently recruiting participants at time for submission of trial protocol 2012-02-10.

### Trial registration

The clinical trial has been registered before the enrollment of the first participant. Date of trial registration: 2011-05-11. Date of first participant randomized: 2011-09-02. ClinicalTrials.gov identifier: NCT01354067. Trial registration has been based on the WHO registration advisory group minimal registration set [82].

## Abbreviations

ACSM, American College of Sports Medicine; CCQ, Clinical COPD questionnaire; CI, confidence interval; CONSORT, consolidate standards of reporting trials; COPD, chronic obstructive pulmonary disease; CPET, cardiopulmonary exercise testing; CRQ, chronic respiratory disease questionnaire; ESES, Exercise Self-Efficacy Scale; HADs, Hospital Anxiety and Depression Scale; HRSLE, high-repetitive single limb exercise; MRC, Medical Research Council; MVC, maximal voluntary contraction; MWT, minute walk test; QoL, quality of life; RCT, randomized controlled trial; RM, repetitions maximum; SD, standard deviation; SPSS, Statistical Package for Social Sciences; SLT, single limb training; UULEX, Unsupported Upper Limb Exercise test; Wmax, Watts maximum.

## Competing interests

The authors declare that they have no competing interests.

## Authors’ contributions

AN has made direct and substantial contribution to this work by conceiving and designing the study, training program and trial protocol, writing the trial protocol and approving the final version of the manuscript. BL has made direct and substantial contribution to this work by providing critical revisions that are important for the intellectual content of the protocol and approving the final version of the manuscript. KW has made direct and substantial contribution to this work by providing the project idea, participating in conceiving the study and trial protocol, providing critical revisions that are important for the intellectual content of the protocol and approving the final version of the manuscript. All authors read and approved the final manuscript.

## Authors’ information

AN: RPT, MSc Physiotherapy, BSc Sport Medicine, BSc Sport Science. Doctoral student at the Department of Community Medicine and Rehabilitation, Physiotherapy, Umeå University, 90187 Umeå, Sweden. BL: RPT, Associate Professor at Department of Community Medicine and Rehabilitation, Physiotherapy, Umeå University, 90187 Umeå, Sweden. KW: RPT, Lecturer at Department of Community Medicine and Rehabilitation, Physiotherapy, Umeå University, 90187 Umeå, Sweden, Department of Public Health and Clinical Medicine Division of Medicine, Umeå University.

## Supplementary Material

Additional file 1 **High-repetitive single limb exercise (HRSLE) program.**Received by both physiotherapists leading training intervention (during education) and participants (at first training session) [6,26,27]. Click here for file

Additional file 2 Placement of participants during each exercise. Click here for file

## References

[B1] Rabe KF Hurd S Anzueto A Barnes PJ Buist SA Calverley P Fukuchi Y Jenkins C Rodriguez-Roisin R van Weel C Zielinski J Global strategy for the diagnosis, management, and prevention of chronic obstructive pulmonary disease: GOLD executive summary Am J Respir Crit Care Med 2007 176 532 555 10.1164/rccm.200703-456SO 17507545

[B2] Chronic obstructive pulmonary disease burden http://www.who.int/respiratory/copd/burden/en/

[B3] Langhammer A Johnsen R Gulsvik A Holmen TL Bjermer L Sex differences in lung vulnerability to tobacco smoking Eur Respir J 2003 21 1017 1023 10.1183/09031936.03.00053202 12797498

[B4] Kanner RE Connett JE Altose MD Buist AS Lee WW Tashkin DP Wise RA Gender difference in airway hyperresponsiveness in smokers with mild COPD. The Lung Health Study Am J Respir Crit Care Med 1994 150 956 961 7921469 10.1164/ajrccm.150.4.7921469

[B5] Houchen L Steiner MC Singh SJ How sustainable is strength training in chronic obstructive pulmonary disease? Physiotherapy 2009 95 1 7 10.1016/j.physio.2008.09.004 19627679

[B6] Gosselink R Troosters T Decramer M Peripheral muscle weakness contributes to exercise limitation in COPD Am J Respir Crit Care Med 1996 153 976 980 8630582 10.1164/ajrccm.153.3.8630582

[B7] Serres I Gautier V Varray A Prefaut C Impaired skeletal muscle endurance related to physical inactivity and altered lung function in COPD patients Chest 1998 113 900 905 10.1378/chest.113.4.900 9554623

[B8] Miranda EF Malaguti C Corso SD Peripheral muscle dysfunction in COPD: lower limbs versus upper limbs J Bras Pneumol 2011 37 380 388 10.1590/S1806-37132011000300016 21755195

[B9] Mador MJ Exercise training in patients with COPD: one leg is better than two? Chest 2008 133 337 339 10.1378/chest.07-2381 18252910

[B10] Richardson RS Leek BT Gavin TP Haseler LJ Mudaliar SR Henry R Mathieu-Costello O Wagner PD Reduced mechanical efficiency in chronic obstructive pulmonary disease but normal peak VO2 with small muscle mass exercise Am J Respir Crit Care Med 2004 169 89 96 14500263 10.1164/rccm.200305-627OC

[B11] Bjorgen S Hoff J Husby VS Hoydal MA Tjonna AE Steinshamn S Richardson RS Helgerud J Aerobic high intensity one and two legs interval cycling in chronic obstructive pulmonary disease: the sum of the parts is greater than the whole Eur J Appl Physiol 2009 106 501 507 10.1007/s00421-009-1038-1 19337746

[B12] Dolmage TE Goldstein RS Effects of one-legged exercise training of patients with COPD Chest 2008 133 370 376 10.1378/chest.07-1423 17925417

[B13] Brønstad E Dedichen H Kirkeby-Garstad I Wisløff U Steinshamn S Effects of one-leg exercise training on mitochondrial function in COPD patients Clin Respir J 2011 5 9 10

[B14] Tyni-Lenne R Dencker K Gordon A Jansson E Sylven C Comprehensive local muscle training increases aerobic working capacity and quality of life and decreases neurohormonal activation in patients with chronic heart failure Eur J Heart Fail 2001 3 47 52 10.1016/S1388-9842(00)00087-8 11163735

[B15] O'Shea SD Taylor NF Paratz JD A predominantly home-based progressive resistance exercise program increases knee extensor strength in the short-term in people with chronic obstructive pulmonary disease: a randomised controlled trial Aust J Physiother 2007 53 229 237 10.1016/S0004-9514(07)70003-X 18047457

[B16] Taylor JD Fletcher JP Reliability of the 8-repetition maximum test in men and women J Sci Med Sport 2012 15 69 73 10.1016/j.jsams.2011.07.002 21820961

[B17] Miranda F Simao R Rhea M Bunker D Prestes J Leite RD Miranda H de Salles BF Novaes J Effects of linear vs. daily undulatory periodized resistance training on maximal and submaximal strength gains J Strength Cond Res 2011 25 1824 1830 10.1519/JSC.0b013e3181e7ff75 21499134

[B18] Moher D Hopewell S Schulz KF Montori V Gotzsche PC Devereaux PJ Elbourne D Egger M Altman DG CONSORT 2010 Explanation and Elaboration: updated guidelines for reporting parallel group randomised trials J Clin Epidemiol 2010 63 e1 e37 Erratum 2012, 65:351 10.1016/j.jclinepi.2010.03.004 20346624

[B19] Boutron I Moher D Altman DG Schulz KF Ravaud P Extending the CONSORT statement to randomized trials of nonpharmacologic treatment: explanation and elaboration Ann Intern Med 2008 148 295 309 18283207 10.7326/0003-4819-148-4-200802190-00008

[B20] Borg GA Perceived exertion: a note on "history" and methods Med Sci Sports 1973 5 90 93 4721012

[B21] Thompson WRG Neil F Pescatello Linda S: ACSM's guidelines for exercise testing and prescription 2010 8 Lippincott Williams & Wilkins, Philadelphia PA

[B22] Bernard S LeBlanc P Whittom F Carrier G Jobin J Belleau R Maltais F Peripheral muscle weakness in patients with chronic obstructive pulmonary disease Am J Respir Crit Care Med 1998 158 629 634 9700144 10.1164/ajrccm.158.2.9711023

[B23] Man WD Soliman MG Gearing J Radford SG Rafferty GF Gray BJ Polkey MI Moxham J Symptoms and quadriceps fatigability after walking and cycling in chronic obstructive pulmonary disease Am J Respir Crit Care Med 2003 168 562 567 10.1164/rccm.200302-162OC 12829456

[B24] Gosker HR Lencer NH Franssen FM van der Vusse GJ Wouters EF Schols AM Striking similarities in systemic factors contributing to decreased exercise capacity in patients with severe chronic heart failure or COPD Chest 2003 123 1416 1424 10.1378/chest.123.5.1416 12740256

[B25] Neptune RR Zajac FE Kautz SA Muscle force redistributes segmental power for body progression during walking Gait Posture 2004 19 194 205 10.1016/S0966-6362(03)00062-6 15013508

[B26] Santiworakul A Jarungjitaree S Jalayondeja W Chantarothorn S Supaibulpipat S Effect of lower extremity exercise on muscle strength and physical capacity in COPD patients J Med Assoc Thai 2009 92 556 563 19374309

[B27] Escamilla RF Yamashiro K Paulos L Andrews JR Shoulder muscle activity and function in common shoulder rehabilitation exercises Sports Med 2009 39 663 685 10.2165/00007256-200939080-00004 19769415

[B28] Page PA Labbe A Topp RV Clinical force production of thera-band elastic bands J Orthop Sports Phys Ther 2000 30 47 48

[B29] Progression models in resistance training for healthy adults Med Sci Sports Exerc 2009 41 687 708 10.1249/MSS.0b013e3181915670 19204579

[B30] Ebben WP Kindler AG Chirdon KA Jenkins NC Polichnowski AJ Ng AV The effect of high-load vs. high-repetition training on endurance performance J Strength Cond Res 2004 18 513 517 15320677 10.1519/R-12722.1

[B31] Campos GE Luecke TJ Wendeln HK Toma K Hagerman FC Murray TF Ragg KE Ratamess NA Kraemer WJ Staron RS Muscular adaptations in response to three different resistance-training regimens: specificity of repetition maximum training zones Eur J Appl Physiol 2002 88 50 60 10.1007/s00421-002-0681-6 12436270

[B32] Janaudis-Ferreira T Wadell K Sundelin G Lindstrom B Thigh muscle strength and endurance in patients with COPD compared with healthy controls Respir Med 2006 100 1451 1457 10.1016/j.rmed.2005.11.001 16337114

[B33] Horowitz MB Littenberg B Mahler DA Dyspnea ratings for prescribing exercise intensity in patients with COPD Chest 1996 109 1169 1175 10.1378/chest.109.5.1169 8625662

[B34] Mercken EM Gosker HR Rutten EP Wouters EF Bast A Hageman GJ Schols AM Systemic and pulmonary oxidative stress after single-leg exercise in COPD Chest 2009 136 1291 1300 10.1378/chest.08-2767 19696125

[B35] Andersen LL Andersen CH Mortensen OS Poulsen OM Bjornlund IB Zebis MK Muscle activation and perceived loading during rehabilitation exercises: comparison of dumbbells and elastic resistance Phys Ther 2010 90 538 549 10.2522/ptj.20090167 20133444

[B36] Page P Ellenbecker ST The Scientific and Clinical Application of Elastic Resistance 2003 Human Kinetics, Champaign, IL

[B37] Newsam C Leese C Fernandez-Silva J Intratester reliability for determing an 8-repetition maximum for 3 shoulder exercises using elastic bands J Sport Rehabilitation 2005 14 35 47

[B38] American College of Sports Medicine Position Stand. The recommended quantity and quality of exercise for developing and maintaining cardiorespiratory and muscular fitness, and flexibility in healthy adults Med Sci Sports and Exercise 1998 30 975 991 10.1097/00005768-199806000-00032 9624661

[B39] Patterson RM Stegink Jansen CW Hogan HA Nassif MD Material properties of Thera-Band Tubing Phys Ther 2001 81 1437 1445 11509073 10.1093/ptj/81.8.1437

[B40] Ries AL Kaplan RM Myers R Prewitt LM Maintenance after pulmonary rehabilitation in chronic lung disease: a randomized trial Am J Respir Crit Care Med 2003 167 880 888 10.1164/rccm.200204-318OC 12505859

[B41] Brooks D Krip B Mangovski-Alzamora S Goldstein RS The effect of post-rehabilitation programmes among individuals with chronic obstructive pulmonary disease Eur Respir J 2002 20 20 29 10.1183/09031936.02.01852001 12166571

[B42] Miller MR Hankinson J Brusasco V Burgos F Casaburi R Coates A Crapo R Enright P van der Grinten CP Gustafsson P Jensen R Johnson DC MacIntyre N McKay R Navajas D Pedersen OF Pellegrino R Viegi G Wanger J Standardisation of spirometry Eur Respir J 2005 26 319 338 10.1183/09031936.05.00034805 16055882

[B43] Wanger J Clausen JL Coates A Pedersen OF Brusasco V Burgos F Casaburi R Crapo R Enright P van der Grinten CP Gustafsson P Hankinson J Jensen R Johnson D Macintyre N McKay R Miller MR Navajas D Pellegrino R Viegi G Standardisation of the measurement of lung volumes Eur Respir J 2005 26 511 522 10.1183/09031936.05.00035005 16135736

[B44] Clinical exercise testing with reference to lung diseases: indications, standardization and interpretation strategies. ERS Task Force on Standardization of Clinical Exercise Testing Eur Respir J 1997 10 2662 2689 9426113 10.1183/09031936.97.10112662

[B45] Ferrazza AM Martolini D Valli G Palange P Cardiopulmonary exercise testing in the functional and prognostic evaluation of patients with pulmonary diseases Respiration 2009 77 3 17 10.1159/000186694 19145106

[B46] Borg GA Psychophysical bases of perceived exertion Med Sci Sports Exerc 1982 14 377 381 7154893

[B47] Jensen D Amjadi K Harris-McAllister V Webb KA O'Donnell DE Mechanisms of dyspnoea relief and improved exercise endurance after furosemide inhalation in COPD Thorax 2008 63 606 613 10.1136/thx.2007.085993 18250181

[B48] Martinez JA Straccia L Sobrani E Silva GA Vianna EO Filho JT Dyspnea scales in the assessment of illiterate patients with chronic obstructive pulmonary disease Am J Med Sci 2000 320 240 243 10.1097/00000441-200010000-00003 11061348

[B49] Zhan S Cerny FJ Gibbons WJ Mador MJ Wu YW Development of an unsupported arm exercise test in patients with chronic obstructive pulmonary disease J Cardiopulm Rehabil 2006 26 180 187 Discussion 188–190 10.1097/00008483-200605000-00013 16738459

[B50] Bauldoff GS Rittinger M Nelson T Doehrel J Diaz PT Feasibility of distractive auditory stimuli on upper extremity training in persons with chronic obstructive pulmonary disease J Cardiopulm Rehabil 2005 25 50 55 10.1097/00008483-200501000-00011 15714113

[B51] Janaudis-Ferreira T Hill K Goldstein RS Robles-Ribeiro P Beauchamp MK Dolmage TE Wadell K Brooks D Resistance arm training in patients with chronic obstructive pulmonary disease: a randomized controlled trial Chest 2011 139 151 158 10.1378/chest.10-1292 20724740

[B52] Takahashi T Jenkins SC Strauss GR Watson CP Lake FR A new unsupported upper limb exercise test for patients with chronic obstructive pulmonary disease J Cardiopulm Rehabil 2003 23 430 437 10.1097/00008483-200311000-00007 14646791

[B53] Wise RA Brown CD Minimal clinically important differences in the six-minute walk test and the incremental shuttle walking test COPD: J Chronic Obstructive Pulmonary Dis 2005 2 125 129 10.1081/COPD-200050527 17136972

[B54] Costi S Crisafulli E Antoni FD Beneventi C Fabbri LM Clini EM Effects of unsupported upper extremity exercise training in patients with COPD: a randomized clinical trial Chest 2009 136 387 395 10.1378/chest.09-0165 19567487

[B55] ATS statement: guidelines for the six-minute walk test Am J Respir Crit Care Med 2002 166 111 117 12091180 10.1164/ajrccm.166.1.at1102

[B56] Lindström BW Sundelin G Ahlgren C Test-retest reliability of biomechanical output and subjective ratings of exertion in isometric and isokinetic shoulder forward flexion in healthy adults Adv Physiother 2003 5 169 178 10.1080/14038190310017606

[B57] Vieira L Bottaro M Celes R Viegas CA E-Silva CA Isokinetic muscle evaluation of quadriceps in patients with chronic obstructive pulmonary disease Rev Port Pneumol 2010 16 717 736 20927491

[B58] Silva KR Marrara KT Marino DM Di Lorenzo VAP Jamami M Skeletal muscle weakness and exercise intolerance in patients with chronic obstructive pulmonary disease [in Portuguese] Revista Brasileira De Fisioterapia 2008 12 169 175

[B59] Malaguti C Napolis LM Villaca D Neder JA Nery LE Dal-Corso S Relationship between peripheral muscle structure and function in patients with chronic obstructive pulmonary disease with different nutritional status J Strength Conditioning Res 2011 25 1795 1803 10.1519/JSC.0b013e3181e501c1 21490512

[B60] Roy JS Ma B Macdermid JC Woodhouse LJ Shoulder muscle endurance: the development of a standardized and reliable protocol Sports Med Arthrosc Rehabil Ther Technol 2011 3 1 10.1186/1758-2555-3-1 21223588 PMC3025902

[B61] Larsson K COPD Chronic Obstructive Pulmonary Disease [in Swedish] 2002 Boehringer Ingelheim AB, Stockholm

[B62] Beauchamp MK Hill K Goldstein RS Janaudis-Ferreira T Brooks D Impairments in balance discriminate fallers from non-fallers in COPD Respir Med 2009 103 1885 1891 10.1016/j.rmed.2009.06.008 19592229

[B63] Kovelis D Segretti NO Probst VS Lareau SC Brunetto AF Pitta F Validation of the Modified Pulmonary Functional Status and Dyspnea Questionnaire and the Medical Research Council scale for use in Brazilian patients with chronic obstructive pulmonary disease J Bras Pneumol 2008 34 1008 1018 10.1590/S1806-37132008001200005 19180335

[B64] Guyatt GH Berman LB Townsend M Pugsley SO Chambers LW A measure of quality of life for clinical trials in chronic lung disease Thorax 1987 42 773 778 10.1136/thx.42.10.773 3321537 PMC460950

[B65] Goldstein RS Gort EH Stubbing D Avendano MA Guyatt GH Randomised controlled trial of respiratory rehabilitation Lancet 1994 344 1394 1397 10.1016/S0140-6736(94)90568-1 7968075

[B66] van der Molen T Willemse BW Schokker S ten Hacken NH Postma DS Juniper EF Development, validity and responsiveness of the Clinical COPD Questionnaire Health Qual Life Outcomes 2003 1 13 10.1186/1477-7525-1-13 12773199 PMC156640

[B67] Stallberg B Nokela M Ehrs PO Hjemdal P Jonsson EW Validation of the clinical COPD Questionnaire (CCQ) in primary care Health Qual Life Outcomes 2009 7 26 10.1186/1477-7525-7-26 19320988 PMC2666647

[B68] Sullivan M Karlsson J Ware J The Swedish SF-36 health survey - I. Evaluation of data quality, scaling assumptions, reliability and construct validity across general populations in Sweden Soc Sci Med 1995 41 1349 1358 10.1016/0277-9536(95)00125-Q 8560302

[B69] Lewis CA Fergusson W Eaton T Zeng I Kolbe J Isolated nocturnal desaturation in COPD: prevalence and impact on quality of life and sleep Thorax 2009 64 133 138 10.1136/thx.2007.088930 18390630

[B70] McCarthy MJ Grevitt MP Silcocks P Hobbs G The reliability of the Vernon and Mior neck disability index, and its validity compared with the short form-36 health survey questionnaire Eur Spine J 2007 16 2111 2117 10.1007/s00586-007-0503-y 17922152 PMC2140132

[B71] Zigmond AS Snaith RP The hospital anxiety and depression scale Acta Psychiatr Scand 1983 67 361 370 10.1111/j.1600-0447.1983.tb09716.x 6880820

[B72] Hellstrom K Vahlberg B Urell C Emtner M Fear of falling, fall-related self-efficacy, anxiety and depression in individuals with chronic obstructive pulmonary disease Clin Rehabil 2009 23 1136 1144 10.1177/0269215509342329 19906765

[B73] Kroll T Kehn M Ho PS Groah S The SCI Exercise Self-Efficacy Scale (ESES): development and psychometric properties Int J Behav Nutr Phys Act 2007 4 34 10.1186/1479-5868-4-34 17760999 PMC2034591

[B74] Davis AH Carrieri-Kohlman V Janson SL Gold WM Stulbarg MS Effects of treatment on two types of self-efficacy in people with chronic obstructive pulmonary disease J Pain Symptom Manage 2006 32 60 70 10.1016/j.jpainsymman.2006.01.012 16824986

[B75] Lacasse Y Martin S Lasserson TJ Goldstein RS Meta-analysis of respiratory rehabilitation in chronic obstructive pulmonary disease. A Cochrane systematic review Eura Medicophys 2007 43 475 485 18084170

[B76] Vierron E Giraudeau B Design effect in multicenter studies: gain or loss of power? BMC Med Res Methodol 2009 9 39 10.1186/1471-2288-9-39 19538744 PMC2715424

[B77] Vierron E Giraudeau B Sample size calculation for multicenter randomized trial: taking the center effect into account Contemp Clin Trials 2007 28 451 458 10.1016/j.cct.2006.11.003 17188941

[B78] O'Shea SD Taylor NF Paratz J Peripheral muscle strength training in COPD: a systematic review Chest 2004 126 903 914 10.1378/chest.126.3.903 15364773

[B79] Skumlien S Skogedal EA Bjortuft O Ryg MS Four weeks' intensive rehabilitation generates significant health effects in COPD patients Chron Respir Dis 2007 4 5 13 10.1177/1479972306070374 17416147

[B80] Krleza-Jeric K Lemmens T 7th revision of the Declaration of Helsinki: good news for the transparency of clinical trials Croat Med J 2009 50 105 110 10.3325/cmj.2009.50.105 19399942 PMC2681053

[B81] Richardson RS Sheldon J Poole DC Hopkins SR Ries AL Wagner PD Evidence of skeletal muscle metabolic reserve during whole body exercise in patients with chronic obstructive pulmonary disease Am J Respir Crit Care Med 1999 159 881 885 10051266 10.1164/ajrccm.159.3.9803049

[B82] De Angelis C Drazen JM Frizelle FA Haug C Hoey J Horton R Kotzin S Laine C Marusic A Overbeke AJ Schroeder TV Sox HC Van Der Weyden MB Clinical trial registration: a statement from the International Committee of Medical Journal Editors Am J Phys Med Rehabil 2005 84 3 4 10.1097/01.PHM.0000150789.89557.43 15632482

