# Supplementary material for: Assessing the effect of high-repetitive single limb exercises (HRSLE) on exercise capacity and quality of life in patients with chronic obstructive pulmonary disease (COPD): study protocol for randomized controlled trial
Source: Trials. 2012 Jul 23;13:114. doi: 10.1186/1745-6215-13-114 (PMC3443039; doi:10.1186/1745-6215-13-114)
Supplement: Additional file 1 — High-repetitive single limb exercise (HRSLE) program.Received by both physiotherapists leading training intervention (during education) and participants (at first training session) [62627]. [file 1745-6215-13-114-S1.doc]

Appendix 1

| **Single limb exercise regimen.** 3 session per week, for 8 weeks (Totally 24 sessions**) Duration:** Warm-up 10 min**,** exercises 40 min and cool-down 10 min. | | | | | |
| --- | --- | --- | --- | --- | --- |
| **Exercise** | **Illustration**  (Start – end. position) | | **Involved muscle**  (Anterior / Posterior) | **Information**  (Set, repetitions, rest and speed) | **Execution**  (Placement of elastic resistance, position and how to perform the exercise) |
| Latissimus row. | |  |  | **Set**: 2 sets per arm  **Repetitions**: 25  **Rest**: When exercising right arm, left arm rests and vice versa.  **Speed**: 1 second when pulling down and 1 second returning arm to start position. | Sit/stand facing the insertion of the elastic band at the red cone with the arm fully extended (90° in shoulder). Keep a firm grip on the elastic band and pull your arm backwards/down towards your hip. End position is with your arm straight down (0° in all directions). Raise your arm with the same speed to start position [27].  **Keep in mind:** Keep your arm straight throughout the movement and use your back and shoulder muscles. |
| Heel raise | |  |  | **Set**: 2 sets per leg  **Repetitions**: 25  **Rest**: When exercising right leg, left leg rests and vice versa.  **Speed**: 1 second when lifting heel of the floor and 1 second returning heel to start position. | Stand facing the wall with one foot flat on the floor, the other kept of the floor. If you need support, steady yourself with your hand against the wall bars. Stand on one leg keeping it straight. Lift your heel of the floor so you´re standing on your toes. Look straight forward avoiding looking down. Lower the heel with the same speed to the start position [26].  **Keep in mind:** Do as many as you can on one leg, if you can’t perform 25 repetitions proceed with two legs. If you can’t reach 25 repetitions with two legs you rest until the next set starts. Start the next set with one leg again. |
| Chest press | |  |  | **Set**: 2 sets per arm  **Repetitions**: 25  **Rest**: When exercising right arm, left arm rests and vice versa.  **Speed**: 1 second when pushing forward and 1 second returning arm to start position. | Sit/stand with your back facing the insertion for the elastic band and your arm abducted at the blue cone. Your hand should be facing forward with the back of the hand upwards. Press your arm forward until its fully extended, mark your end position and release in a controlled manner your arm back to the start position with the same speed [27].  **Keep in mind**: Keep your lower back and abdominal muscles in tension throughout the whole exercise. |
| Leg extension | |  |  | **Set**: 2 sets per leg  **Repetitions**: 25  **Rest**: When exercising right leg, left leg rests and vice versa.  **Speed**: 1 second when extending and 1 second returning leg to start position. | Sit with your back facing the insertion for the elastic band at orange cone. Starting position with your knee and hip both in 90°. Fasten the elastic band around your ankle. Fully extend your leg then release in a controlled manner to the start position with the same speed [6].  **Keep in mind:** Fully extend your knee at every repetition |
| Illustrations reproduced with permission. Copyright ©2011 Exercise Organizer AS | | | | | |
| **Exercise** | **Illustration**  (Start – end. position) | | **Involved muscle**  (Anterior / Posterior) | **Information**  (Set, repetitions, rest and speed) | **Execution**  (Placement of elastic resistance, position and how to perform the exercise) |
| Straight arm shoulder flexion | |  |  | **Set**: 2 sets per arm  **Repetitions**: 25  **Rest**: When exercising right arm, left arm rests and vice versa.  **Speed**: 1 second when lifting arm upwards and 1 second returning arm to start position. | Sit/stand with your back facing the insertion for the elastic band at the yellow cone. You arm kept straight holding the band in full can position with thumb pointing upwards. Lift your arm upwards/forward until your elbow is in line with your forehead [27]. Then release in a controlled manner to the start position with the same speed.  **Keep in mind:** Keep your arm fully extended throughout the whole movement |
| Leg curl | | **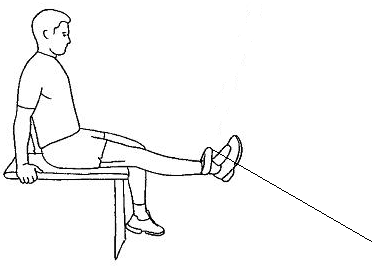** 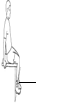 |  | **Set**: 2 sets per leg  **Repetitions**: 25  **Rest**: When exercising right leg, left leg rests and vice versa.  **Speed**: 1 second when flexing knee and 1 second returning leg to start position. | Sit facing the insertion for the elastic band at the blue cone. Fasten the elastic band around your ankle. With the exercising leg pointing straight forward, then bend your knee pulling your foot down/inwards under the stool as far as you can. Then release in a controlled manner to the start position with the same speed.  **Keep in mind:** Flex as much as possible in every repetition |
| Elbow flexion | |  |  | **Set**: 2 sets per arm  **Repetitions**: 25  **Rest**: When exercising right arm, left arm rests and vice versa.  **Speed**: 1 second when flexing arm and 1 second returning arm to start position. | Sit/stand facing the insertion for the elastic band at the white cone with your arm kept straight in the direction of the elastic band. Hold your arm against your body. Move the elastic band upwards by flexing your elbow until the palm of your hand points towards your chest. End position when maximal flexion in elbow joint. Then release in a controlled manner to the start position with the same speed.  **Keep in mind:** Fixate your elbow against your body throughout the exercise. |
| Step-up | |  |  | **Set**: 2 sets per leg  **Repetitions**: 25  **Rest**: When exercising right leg, left leg rests and vice versa.  **Speed**: 1-3 second when stepping up and 1-3 stepping down to start position. | Stand facing the step-up board with feet at shoulder width. Step up with right foot on top of the board and place the whole foot on the surface. Then lift left foot upwards and “touch” the step-up board’s surface then lower the foot down to start position, finally step down with right foot to start position with the same speed [26].  **Keep in mind:** Don’t swing, step up on top of the step-up board in a controlled manner. |

Illustrations reproduced with permission. Copyright ©2011 Exercise Organizer AS
