# Supplementary material for: Assessing the effect of high-repetitive single limb exercises (HRSLE) on exercise capacity and quality of life in patients with chronic obstructive pulmonary disease (COPD): study protocol for randomized controlled trial
Source: Trials. 2012 Jul 23;13:114. doi: 10.1186/1745-6215-13-114 (PMC3443039; doi:10.1186/1745-6215-13-114)
Supplement: Additional file 2 — Placement of participants during each exercise. [file 1745-6215-13-114-S2.doc]

Appendix 2

The illustration shows start position for each exercise, calculation of strength relationship is demonstrated for end positions.

**Position of cones based on percent difference in strength between muscles in each exercise**

Straight arm shoulder Latissimus row Elbow flexion Chest press Leg extension

flexion Leg curl

1.25m

1.875m

2.625m

2.25m

3m

**Meters from insertion**

1m 1.25m 1.5m 1.75m 2m 2.25m 2.5m 2.75m 3m

**Percent stretch of elastic resistance band (relaxed length 1 meter)**

0% 25% 50% 75% 100% 125% 150% 175% 200%

**Approximately** **percent change in weight at a given extension of the elastic resistance. Start point at 200% elongation.**

-70% -60% -50% -40% -30% -20% -10% 0
